# Immune cell composition in normal human kidneys

**DOI:** 10.1038/s41598-020-72821-x

**Published:** 2020-09-24

**Authors:** Jun-Gyu Park, Myeongsu Na, Min-Gang Kim, Su Hwan Park, Hack June Lee, Dong Ki Kim, Cheol Kwak, Yon Su Kim, Sunghoe Chang, Kyung Chul Moon, Dong-Sup Lee, Seung Seok Han

**Affiliations:** 1 Department of Biomedical Sciences, Seoul National University College of Medicine, 103 Daehakro, Jongno-gu, Seoul, 03080 South Korea; 2 Department of Internal Medicine, Seoul National University College of Medicine, 103 Daehakro, Jongno-gu, Seoul, 03080 South Korea; 3 Department of Urology, Seoul National University College of Medicine, 103 Daehakro, Jongno-gu, Seoul, 03080 South Korea; 4 Department of Pathology, Seoul National University College of Medicine, 103 Daehakro, Jongno-gu, Seoul, 03080 South Korea

**Keywords:** Translational immunology, Nephrology

## Abstract

An understanding of immunological mechanisms in kidney diseases has advanced using mouse kidneys. However, the profiling of immune cell subsets in human kidneys remains undetermined, particularly compared with mouse kidneys. Normal human kidneys were obtained from radically nephrectomised patients with urogenital malignancy (n = 15). Subsequently, human kidney immune cell subsets were analysed using multicolor flow cytometry and compared with subsets from C57BL/6 or BALB/c mice under specific pathogen-free conditions. Twenty kidney sections from healthy kidney donors or subjects without specific renal lesions were additionally analysed by immunohistochemistry. In human kidneys, 47% ± 12% (maximum 63%) of immune cells were CD3^+^ T cells. Kidney CD4^+^ and CD8^+^ T cells comprised 44% and 56% of total T cells. Of these, 47% ± 15% of T cells displayed an effector memory phenotype (CCR7^−^ CD45RA^−^ CD69^−^), and 48% ± 19% were kidney-resident cells (CCR7^−^ CD45RA^−^ CD69^+^). However, the proportions of human CD14^+^ and CD16^+^ myeloid cells were approximately 10% of total immune cells. A predominance of CD3^+^ T cells and a low proportion of CD14^+^ or CD68^+^ myeloid cells were also identified in healthy human kidney sections. In mouse kidneys, kidney-resident macrophages (CD11b^low^ F4/80^high^) were the most predominant subset (up to 50%) but the proportion of CD3^+^ T cells was less than 20%. These results will be of use in studies in which mouse results are translated into human cases under homeostatic conditions or with disease.

## Introduction

Understanding immune dysregulation in kidney diseases has been advanced recently because of sophisticated mouse studies^1^. Chronic kidney disease, acute kidney injury, end-stage renal disease, or even genetic disorders are directly or indirectly immune-mediated^2,3^, and thus several immune-modulating agents have been investigated in clinical trials^4^. Current options are restricted to conventional systemic agents such as glucocorticoids, calcineurin inhibitors, and mycophenolic acid or non-immune-modulating agents, including angiotensin converting enzyme inhibitor and angiotensin II receptor blocker^5,6^. Targeting agents such as Rituximab and Belatacept have been used in certain kidney diseases such as lupus nephritis, vasculitis, and kidney transplantation^7,8^. Despite the efficacy of these agents, further immune-cell-targeting agents are needed to improve patient outcomes and decrease systemic side effects.

To accomplish this, investigations of the renal immune system should not be restricted to mice but be expanded to humans because there may be potential differences between these species^9^. In the blood, neutrophils are the predominant subset in humans (70%), whereas lymphocytes are predominant in mice (up to 90%)^10^. A different ratio of lymphoid to myeloid cells is thought to exist in peripheral tissues^11^, but this has not been investigated thoroughly because it is difficult to obtain normal peripheral tissues with sufficient volume to be analysed. Recent studies revealed that certain resident immune cells are predominant in human peripheral tissues such as the brain and lungs^12,13^, but information on resident immune cells in human kidneys is lacking^14^. Macrophages are the main immune cell subset in the kidneys of mice bred under specific pathogen-free conditions^15^, but the main subset in human kidneys remains unresolved. Analysis of predominant immune cell subsets in human tissues may enhance our understanding of homeostasis, inflammation, and disease, particularly in the kidneys, because some kidney diseases are immune-mediated. The present study investigated whether the ratio of lymphocytes to myeloid cells is similar between human and mouse kidneys, the former of which were obtained from patients who underwent radical nephrectomy. The results showed that human kidneys predominantly harboured T cells, especially effector memory and resident memory cell subsets. These results will be of use in studies in which mouse results are translated into human cases under homeostatic conditions or with disease.

## Results

### Immune cell subsets in human kidneys

Fifteen kidney tissues from radical nephrectomy cases were analysed by flow cytometry. The mean age of patients was 69 ± 10 years. Ten patients (66.7%) were male. The mean values of serum creatinine and the estimated glomerular filtration rate calculated by the Chronic Kidney Disease Epidemiology Collaboration equation^16^ were 1.0 ± 0.3 mg/dl and 73.6 ± 15.2 ml/min/1.73 m^2^, respectively. Thirteen patients (86.7%) had estimated glomerular filtration rates greater than 60 ml/min/1.73 m^2^. Three patients (20%) had proteinuria by dipstick test, and all the levels were 1+. Other baseline characteristics are shown in Supplementary Table S1. Histologic findings are shown in Supplementary Table S2.

**Figure 1:**
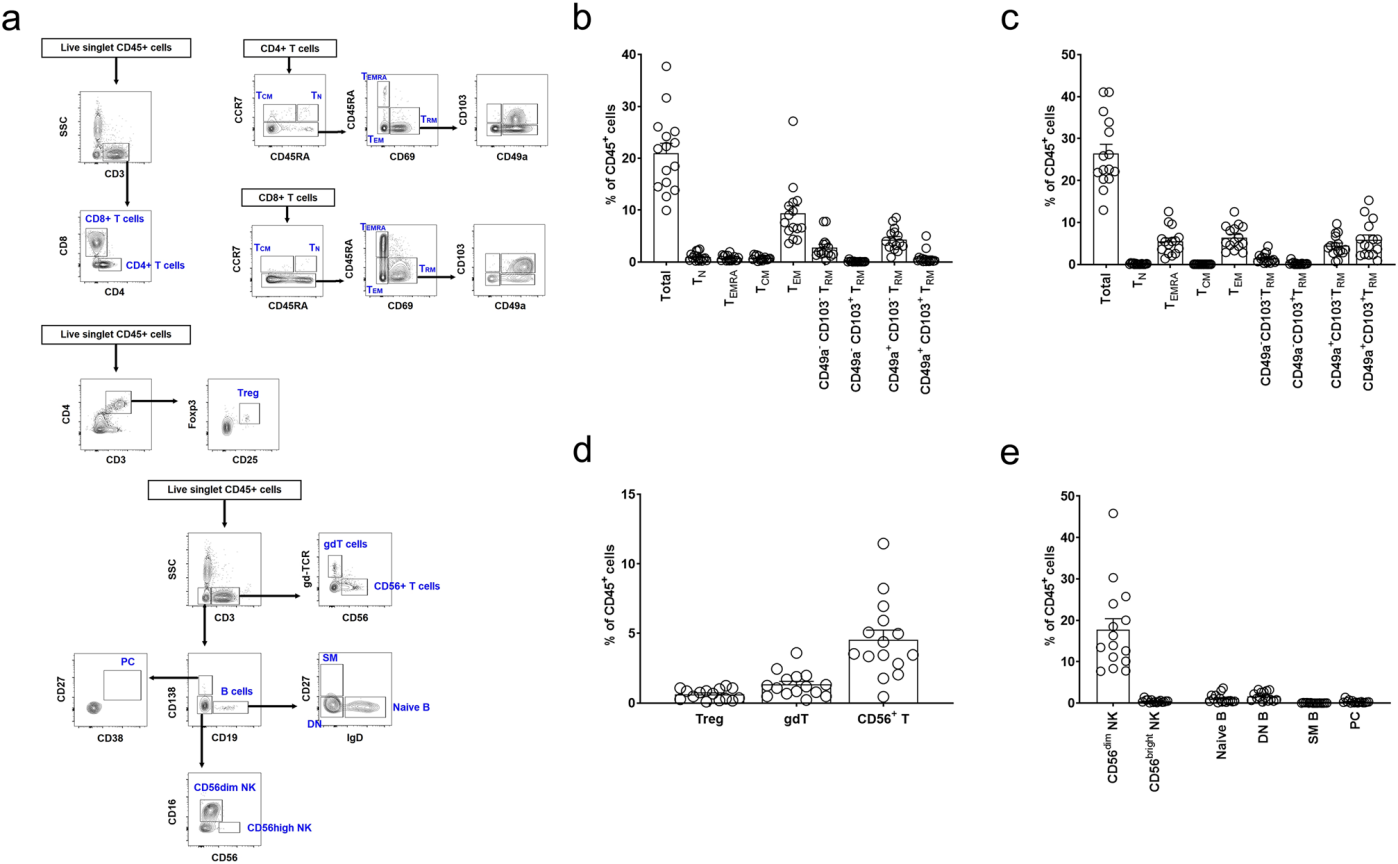
Lymphocytes and natural killer (NK) cells in human kidneys. (**a**) Gating strategy for kidney T cell, B cell, and NK cell subsets. (**b**) Proportion of the CD4^+^ T cell subset. (**c**) Proportion of the CD8^+^ T cell subset. (**d**) Proportions of Treg, gdT, and CD56^+^ T cell subsets. (**e**) Proportions of B cell and NK cell subsets. *T*_*N*_ naïve T, *T*_*CM*_ central memory T, *T*_*EM*_ effector memory T, *T*_*EMRA*_ CD45RA^+^ effector memory T, *T*_*RM*_ resident memory T, *Treg* regulatory T, *gdT* gamma/delta T, *PC* plasma cell, *SM* switched-memory B, *DN* IgD^−^ CD27^−^ B. n = 15.

Flow cytometric analysis of immune cell subsets in kidney tissues from patients who underwent radical nephrectomy was performed. Figure 1a shows the representative gating strategy for T cell, B cell, and natural killer cell subsets. The proportion of CD3^+^ T cells was 47.4% ± 11.6%, of which approximately 44% and 56% were CD4^+^ and CD8^+^ T cells, respectively. The mean ratio of CD4^+^ per CD8^+^ T cells was 0.9 ± 0.4. This low CD4/CD8 ratio was the reverse of values observed in human peripheral blood^17^.

Among CD4^+^ T cells (Fig. 1b), the main subsets were CCR7^−^ CD45RA^−^ cells (effector memory; T_EM_: 44.5% [9.3% of CD45^+^ cells]) and CD69^+^ cells (tissue-resident memory; T_RM_: 39.3% [8.2% of CD45^+^ cells]). Among CD8^+^ T cells (Fig. 1c), the main subsets were T_EM_ (24.3% [6.4% of CD45^+^ cells]), T_RM_ (57.9% [15.3% of CD45^+^ cells]), and CCR7^−^ CD45RA^+^ cells (T_EMRA_) (20.7% [5.5% of CD45^+^ cells]). When we grouped T_RM_ cells by the expression of CD103 and CD49a^18^, CD49a^−^ CD103^−^ and CD49a^+^ CD103^−^ T_RM_ cells were the predominant subsets in CD4^+^ T_RM_ cells, and CD49a^−^ CD103^−^, CD49a^+^ CD103^−^, and CD49a^+^ CD103^+^ T_RM_ subsets were predominant in CD8^+^ T_RM_ cells. However, CD49a^−^ CD103^+^ T_RM_ cells were the minor subset in CD4^+^ and CD8^+^ T_RM_ cells (< 1% of CD45^+^ cells). Regarding other T cell subsets, regulatory T (Treg), gamma/delta (γδ) T, and CD56^+^ T cells were less than 10% of CD45^+^ immune cells (Fig. 1d). The proportions of NK and B cells were 18.2% ± 10.5% and 1.4% ± 1.2%, respectively (Fig. 1e). Among NK cells, the CD56^dim^ subset was the main population. Switched-memory B cells and plasma cells constituted less than 1% of CD45^+^ cells.

**Figure 2:**
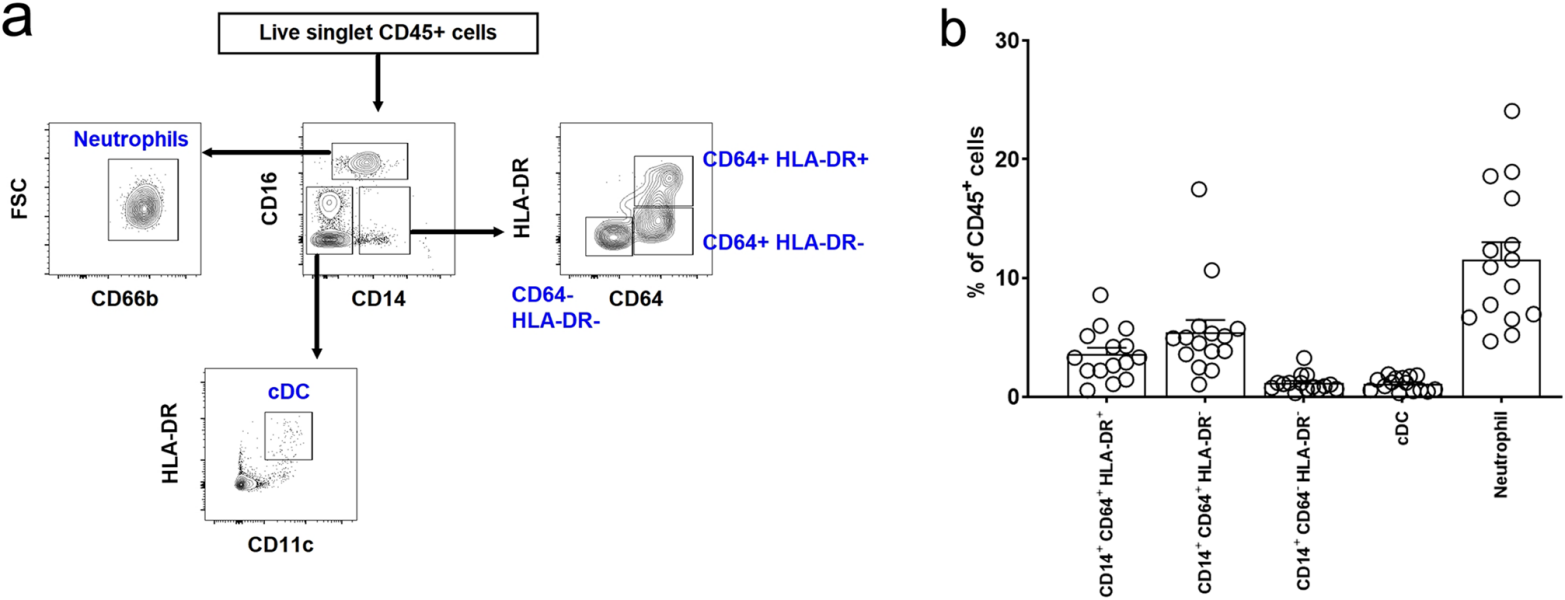
Myeloid cells in human kidneys. (**a**) Gating strategy for kidney monocyte/macrophage, classical dendritic cell (cDC), and neutrophil subsets. (**b**) Proportion of myeloid cell subsets in human kidneys. n = 15.

The gating strategy for myeloid cells including monocytes/macrophages, classical dendritic cells (cDCs), and neutrophils is shown in Fig. 2a. The proportion of the CD14^+^ monocyte/macrophage subset was 10.2% ± 4.7%. Most CD14^+^ monocyte and macrophage subsets in the kidney did not express CD16, and thus, these were categorized by the expression levels of CD64 and HLA-DR^19^. Among CD14^+^ cells, CD64^+^ HLA-DR^+^ (35.1% [3.6% of CD45^+^ cells]) and CD64^+^ HLA-DR^−^ cells (53.6% [5.4% of CD45^+^ cells]) were the main subsets, and CD64^−^ HLA-DR^−^ cells were the minor subset (11.3% [1.2% of CD45^+^ cells]) (Fig. 2b). There were almost no CD64^−^ HLA-DR^+^ cells among CD14^+^ cells. The proportions of cDCs and neutrophils were 1.1% ± 0.6% and 11.5% ± 5.8%, respectively. Collectively, the most abundant immune cell subset in human kidneys was CD3^+^ T cells. This trend did not differ between male and female subjects or was not dependent on kidney dysfunction (see Supplementary Fig. S1).

### Immunostaining analysis of human kidney sections

**Figure 3:**
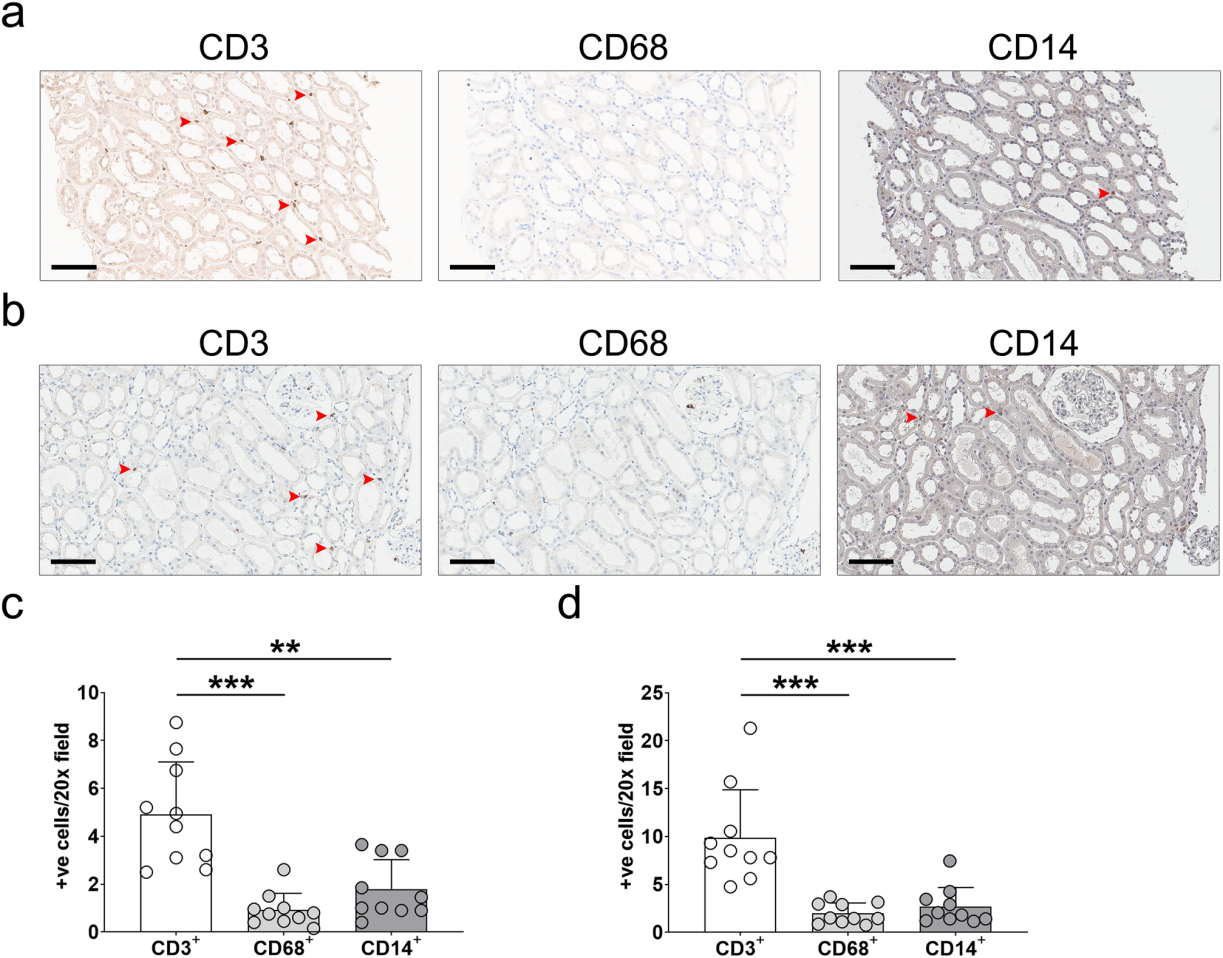
Immunostaining analysis of human kidney sections. (**a**) Representative images of staining for CD3, CD68, and CD14 in healthy kidney donors (n = 10). Scale bar = 10 μm. (**b**) Quantitative analysis of CD3^+^, CD68^+^, and CD14^+^ cells in sections from healthy kidney donors. (**c**) Representative images of staining for CD3, CD68, and CD14 in subjects without specific renal lesions (n = 10). Scale bar = 10 μm. (**d**) Quantitative analysis of CD3^+^, CD68^+^, and CD14^+^ cells in sections from subjects without specific lesions. Arrows indicate representative positive cells. **P < 0.01; ***P < 0.001.

Pre-analytic procedures such as digestion might affect the above flow cytometric results. For sensitivity analysis, kidney sections from healthy donors (i.e., zero-time biopsy) and subjects without specific renal lesions (each n = 10) were evaluated. CD3^+^, CD68^+^, and CD14^+^ cells in the interstitial area were counted after excluding cells within vessels, tubules, and glomeruli. Figure 3a is a representative image of sections from healthy donors. Compared with frequently observed CD3^+^ cells, CD68^+^ or CD14^+^ cells were rarely seen. When stained cells were counted, the number of CD3^+^ cells was higher than those of CD68^+^ and CD14^+^ cells (Fig. 3c). This trend remained consistent in subjects without specific renal lesions (Fig. 3b,d). These results supported the flow cytometric results where CD3^+^ T cells were dominant in human kidneys compared with CD14^+^ monocytes and macrophages. When these interstitial immune cells were stained in normal kidney tissues from nephrectomised patients, the residency of CD3^+^ T cells was higher than that of CD68^+^ or CD14^+^ cells (see Supplementary Fig. S2).

### Immune cell subsets in mouse kidneys

**Figure 4:**
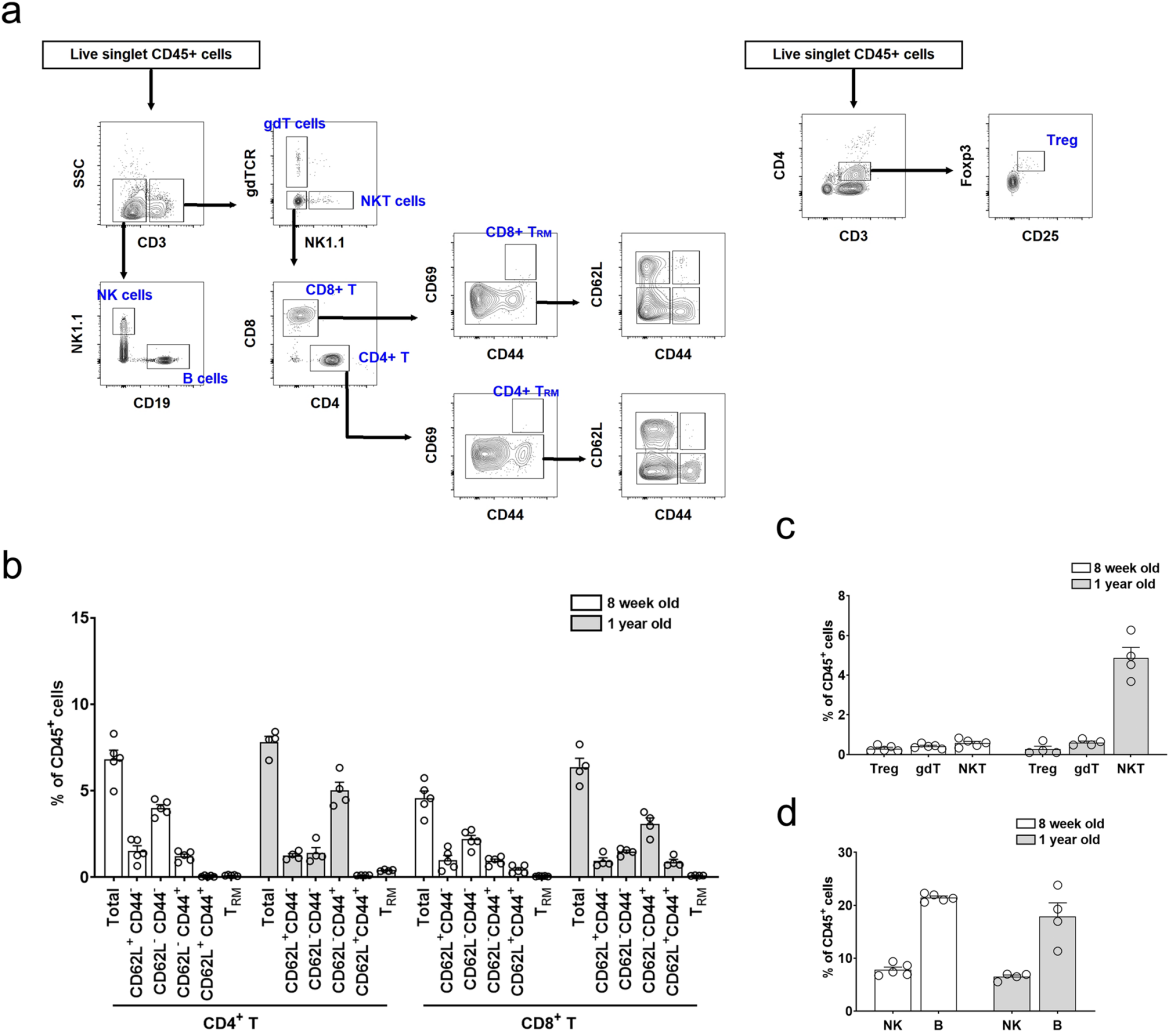
Lymphocytes and natural killer (NK) cells in mouse kidneys. (**a**) Gating strategy for kidney T cell, B cell, and NK cell subsets. (**b**) Proportion of the T cell subset. (**c**) Proportions of Treg, gdT, and NKT cell subsets. (**d**) Proportions of B cell and NK cell subsets. *gdT* gamma/delta T, *T*_*RM*_ resident memory T, *Treg* regulatory T. n = 4–5 per group.

Because human samples were obtained from older patients, 1-year-old mice were analysed in addition to 8-week-old mice. Harvested kidneys from 8-week- and 1-year-old C57BL/6 mice were evaluated after perfusion with 10 ml of cold PBS via the left chamber of the heart. Figure 4a,b show the representative gating strategy for T cells and their proportions. Total CD3^+^ T cells accounted for 11.4% ± 2.1% and 14.2% ± 1.3% in 8-week- and 1-year-old mice, respectively. Among the CD3^+^ T cells in 8-week-old mice, CD4^+^ and CD8^+^ T cells accounted for 60% and 40%, respectively. For 1-year-old mice, CD4^+^ and CD8^+^ T cells accounted for 55% and 45%, respectively. The most abundant T cell subset was CD62L^−^ CD44^−^ cells in 8-week-old mice, whereas CD62L^−^ CD44^+^ cells were most abundant in 1-year-old mice (Fig. 4b). The T_RM_ subset was less than 0.5% of CD45^+^ immune cells. Regarding other T cell subsets, Treg, γδT, and natural killer T (NKT) cells were less than 1% of CD45^+^ immune cells, except in 1-year-old mice where 5% of cells were NKT cells (Fig. 4c). The proportions of NK and B cells were 7.8% ± 1.1% and 21.4% ± 0.7%, respectively, in 8-week-old mice, and 6.5% ± 0.7% and 17.9% ± 5.2% in 1-year-old mice (Fig. 4d).

**Figure 5:**
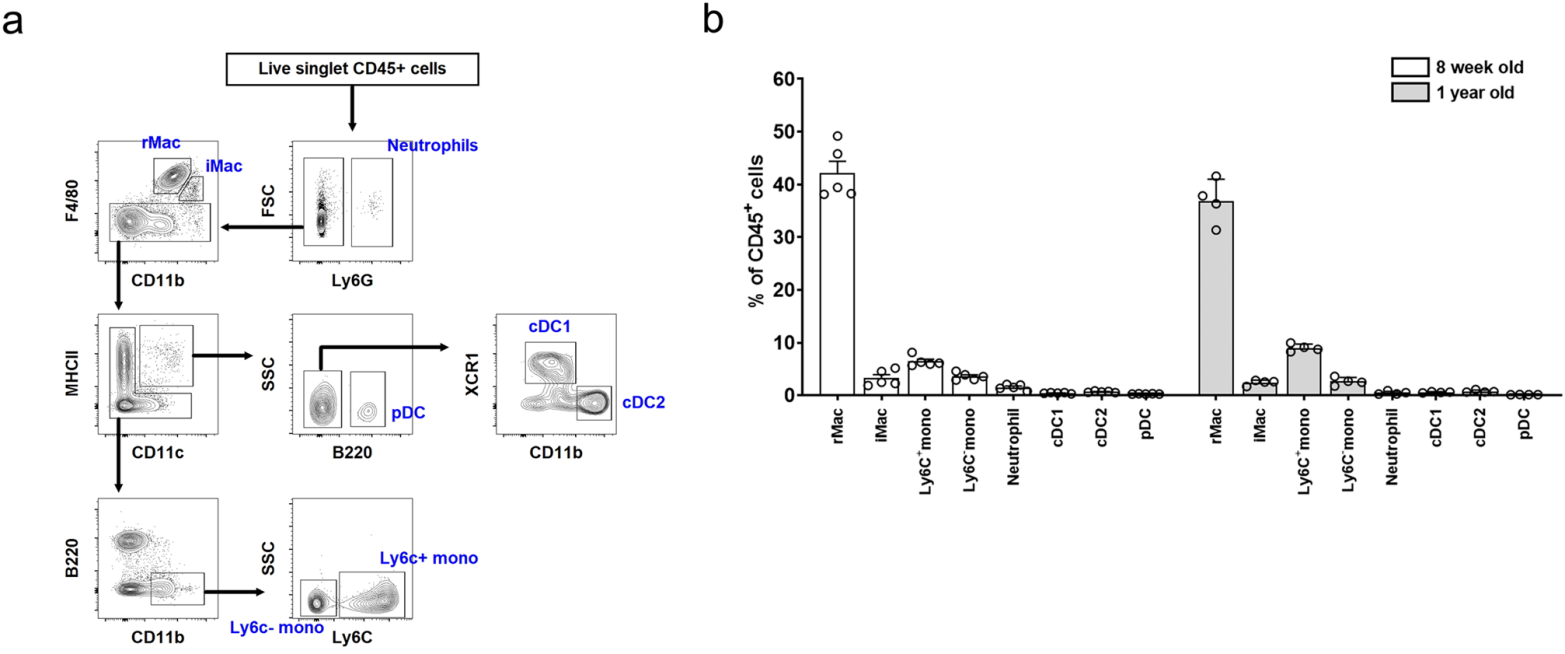
Myeloid cells in mouse kidneys. (**a**) Gating strategy for kidney monocyte (mono), resident and infiltrating macrophages (rMac and iMac, respectively), classical and plasmacytoid dendritic cells (cDCs and pDCs, respectively), and neutrophil subsets. (**b**) Proportion of myeloid cell subsets in mouse kidneys. n = 4–5 per group.

The gating strategy and proportions of macrophages, monocytes, DCs, and neutrophils are shown in Fig. 5a,b. The proportions of macrophages were 45.4% ± 4.0% and 39.2% ± 4.0% in 8-week- and 1-year-old mice, respectively. When macrophages were categorized into kidney-resident (rMac: CD11b^low^ F4/80^high^) and kidney-infiltrating (iMac: CD11b^high^ F4/80^low^) subsets^20,21^, more than 90% were rMac (Fig. 5b). Other myeloid subsets such as neutrophils and dendritic cells were less than 1% of CD45^+^ cells. When 8-week-old BALB/c mice were examined, macrophages were the most abundant subset at 52%, of which more than 90% were rMac (see Supplementary Fig. S3). Collectively, macrophages were the most abundant immune cell subset in the mouse kidney.

When immune subsets were analysed in mice that had not been perfused with PBS, the proportions of macrophages and monocytes were 30.9% ± 4.5% and 12.6% ± 1.1%, respectively in 8-week-old mice, and 26.9% ± 3.1% and 11.8% ± 1.2%, respectively in 1-year-old mice. The proportions of CD3^+^ T cells were 16% and 20% in 8-week- and 1-year-old mice, respectively.

**Figure 6:**
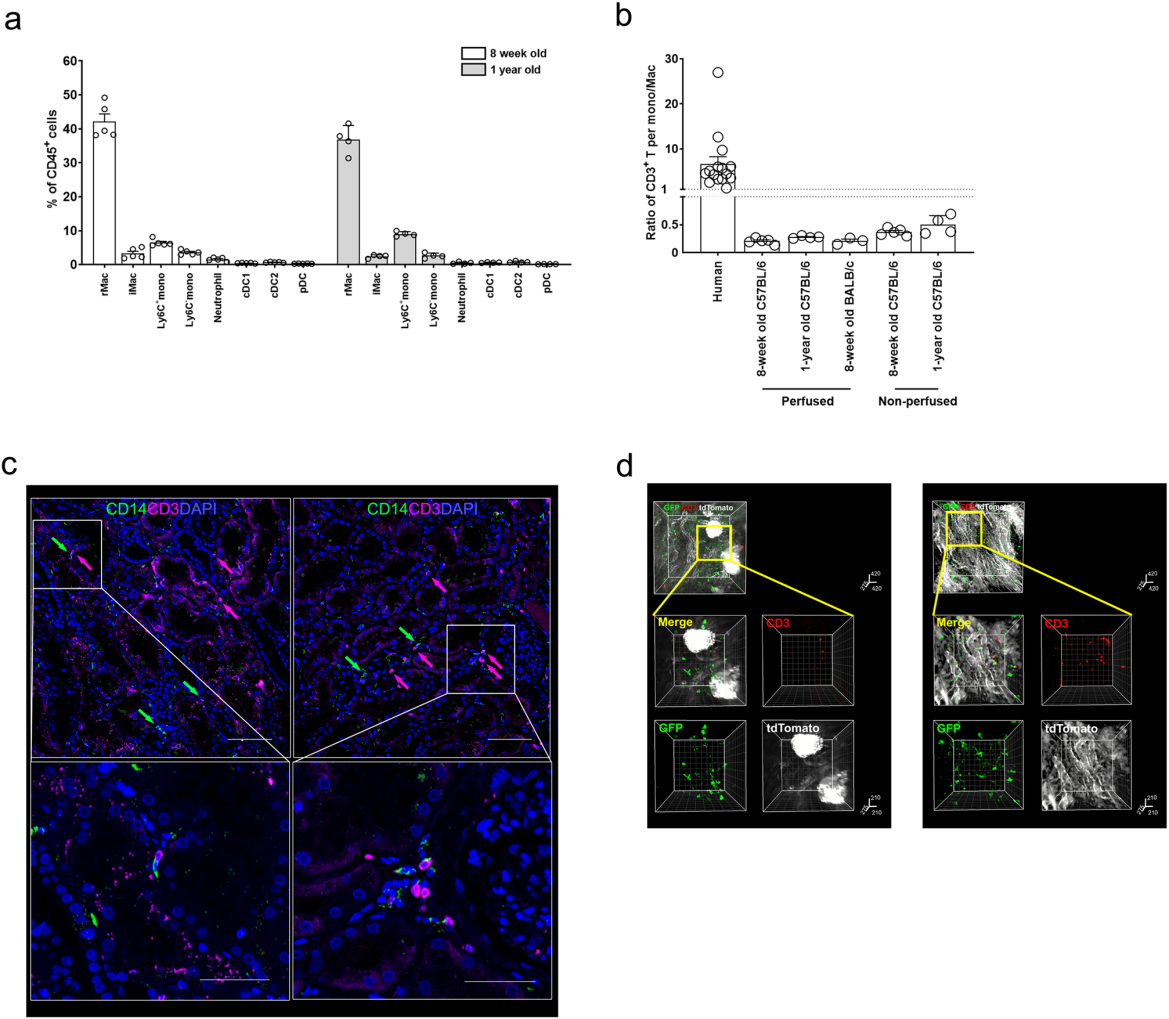
Comparison between human and mouse kidneys. (**a**) Summary bar graph of the proportions of immune cell subsets. Mouse samples represent 8-week old C57BL/6 mice perfused with PBS. *NK* natural killer, *mono* monocyte, *Mac* macrophage. (**b**) Ratio of CD3^+^ T cells to monocytes (mono) and macrophages (Mac) in human and mouse kidneys. (**c**) Two representative images of the immunofluorescence localization of CD3^+^ T cells and CD14^+^ myeloid cells in biopsied kidney tissues from healthy kidney donors (corresponding to Supplementary Video S1). Arrows indicate positively stained cells. Data are representative of two experiments. Scale bar = 100 μm (or 50 μm in zoomed-in images). (**d**) Two representative images of the immunofluorescence localization of CD3^+^ T cells and GFP^+^ macrophages in normal kidneys from *LysM*^Cre^–*ROSA*^mTmG^ mice (corresponding to Supplementary Video S2). Staining was performed after tissue clearing. Data are representative of two experiments.

In summary, the proportion of T cells was higher than that of monocytes/macrophages in human kidneys, in contrast to mouse kidneys (Fig. 6a). Similarly, the ratio of CD3^+^ T cells to monocytes/macrophages was higher in human kidneys than in mouse kidneys (Fig. 6b). Representative immunofluorescence images of human and mouse kidneys show that human kidneys harboured more CD3^+^ T cells than CD14^+^ monocytes/macrophages in contrast to mouse kidneys, which harboured more GFP^+^ monocytes/macrophages (Fig. 6c,d, and Supplementary Videos S1 and S2).

## Discussion

Mouse studies are invaluable for investigating immune-mediated homeostasis and disease, but their translation to human conditions requires further validation because mice, particularly those bred under specific conditions (e.g., specific pathogen-free conditions), might have a different immune and inflammatory milieu compared with humans. The present study investigated whether human kidneys harboured different immune cell subsets from mouse kidneys. Of note, the ratio of CD3^+^ T cells to monocytes/macrophages was higher in human kidneys than in mouse kidneys.

Kidneys have a distinct immune system, and thus, subsets of resident immune cells are different from those in other organs^22,23^. Kidneys contain specialized rMac that monitor and scavenge the endothelial transport of immune complexes, and subsequently trigger type III hypersensitivity responses^20^. These phenomena may be crucial in diseases attributable to the insufficient clearance of immune complexes^24^. The proangiogenic activity of kidney-resident macrophages during renal artery stenosis was also reported^25^. This frontline and potential homeostatic activity of rMac may be related to their high proportion in mouse kidneys. However, the residency and proportion of rMac have not been fully evaluated in human kidneys. Recent single-cell RNA sequencing data suggested that markers such as CD74 and CD81 might characterize human kidney-rMac^26^. However, these markers are also expressed on other myeloid subsets of that dataset or on non-myeloid cells^27^. Accordingly, the present study used conventional myeloid markers such as CD14, CD64, HLA-DR, and CD68 to identify macrophages and monocytes, not rMac alone. Nevertheless, fewer numbers of macrophages and/or monocytes were present in human kidneys compared with mouse kidneys, and this might result in a high frequency of other immune subsets such as T cells in human kidneys.

The present exploratory study did not determine the mechanisms related to the high abundance of T cells within human kidneys. Decreased numbers of other immune cell subsets might lead to an increase in T cells. In addition to this simple hypothesis, we focused on the relatively high proportion of T_RM_. Non-lymphoid tissues such as kidneys harbour certain populations of memory T cells that are not present in the circulation (referred to as T_RM_), which are different from circulating T cells^28^. The cytokine milieu after inflammatory insults, such as infections or ischemic damage, drives the development of T_RM_, and thus, mouse kidneys in the present study had few T_RM_ in the absence of kidney insults. In contrast to mouse conditions, our study subjects might have experienced subclinical injury although most of their kidney functions were within the normal range. Furthermore, continuous contact with uremic toxins, spontaneously-dead parenchymal cells or debris from tubules and glomeruli over the years might affect the development of the T_RM_ subset^29^. The different splenic immune cell profiles between wild and laboratory mice supports the hypothesis that the environmental milieu alters the renal immune cell composition^30^. Intriguingly, CD49a^+^ cells were the predominant CD4^+^ and CD8^+^ T_RM_ subsets, but CD103 was not the primary marker of kidney T_RM_. This feature is different from the phenotype and distribution of T_RM_ subpopulations in other peripheral organs^18^. The characteristics of renal epithelial cells and their adhesion molecules may determine the markers of T_RM_, which will be addressed in another project.

The high proportion of T cells in human kidneys may have implications in clinical situations. The roles of recipient-originated T cells have been much studied, but it is unknown whether effector T cells or T_RM_ cells from kidney donors have a role in the rejection process after kidney transplantation. Although their specificity against recipient antigens may be undetermined or irrelevant, they have the potential to be activated by bystander effects^31^. Long duration of cold ischemia, surgical stress, and other systemic inflammatory cascades after donor death are sufficient to activate donor-resident T cells, which may induce delayed graft function^32^. A previous study reported that donor T cells were associated with the transplant outcome of lungs^33^, and therefore the presence or abundance of donor T cells may be related to kidney transplant outcome. A previous study identified the presence of donor-derived CD8 + T_RM_ cells was associated with graft failure in the first month after transplantation^34^. Additionally, an abundance of T cells may affect the surveillance of kidney cancers because this cancer type is typically immunogenic and T cell-dependent^35^. Possibly because of these characteristics, immunotherapy has emerged as a first-line agent for patients with renal cell carcinoma^36^.

The proportion of renal B cells was much lower than other immune cell subsets such as T cells. These results did not exclude the clinical importance of renal B cells, because many B cells can infiltrate into tissues during disease or inflammation^37^. The intrarenal B-cell infiltrates are associated with outcomes of lupus nephritis, anti-neutrophil cytoplasmic autoantibody vasculitis, and kidney transplants^38–40^. Furthermore, most immune reactions consisting of B cells are evoked within germinal centers in lymphoid organs^41^; however, the present study did not explore the renal lymph nodes. Future studies on inflammation in human kidneys or human renal lymph nodes will clarify the role of tissue-localized B cells.

The popularity of mice has increased in research to understand immune reactions in homeostatic and inflammatory kidneys. Despite the worth of mouse studies, results from these investigations should be taken with caution before translating results to human conditions in which T cells are more abundant. Future studies will determine the reasons for the high abundance of renal T cells and their clinical implications under homeostatic and disease conditions.

## Methods

### Animals

C57BL/6 and BALB/c wild type male mice, and *LysM*^Cre^ and *ROSA*^mTmG^ male mice were purchased from the Jackson Laboratory (ME, USA). The *ROSA*^mTmG^ mouse is a reporter mouse expressing membrane tdTomato in all cells except those induced to express Cre recombinase, which changes reporter expression to membrane GFP. Accordingly, *LysM*^Cre^–*ROSA*^mTmG^ mice display GFP fluorescence in lysozyme^+^ cells, such as macrophages, and tdTomato fluorescence in other cells. Mice were housed under specific pathogen-free conditions at the Seoul National University College of Medicine. All experiments were approved by the Seoul National University Institutional Animal Care and Use Committee (no. SNU-150611-1-19) and in accordance with the guidelines.

### Human kidneys

Human kidney tissues were obtained from patients who underwent radical nephrectomy because of urogenital malignancy (renal cell carcinoma [n = 9], urothelial carcinoma [n = 5], and metastasis of signet ring cell carcinoma [n = 1]), when there was no evidence of hydronephrosis or infectious disease. Normal tissues, including the cortex and medulla, were located at the opposite pole of the tumours, and non-intrusion of the tumour was confirmed by computed tomography imaging or light microscopy. For sensitivity analysis, kidney sections from healthy living donors (i.e., zero-time biopsy) and subjects without specific renal lesions such as glomerulonephritis and tubulointersitital nephritis (each n = 10) were evaluated. The donors did not have hypertension, diabetes mellitus, or other diseases. The study design for human sample use was approved by the institutional review board of the Seoul National University Hospital (no. H-1810-016-975) and complied with the Declaration of Helsinki. All patients provided written informed consent for the donation and use of their specimens in the present study.

### Flow cytometry

Human and mouse kidney tissues were minced and digested with 40 μg/ml DNase I and 1 mg/ml collagenase D for 30–45 min at 37 °C. Cells were filtered through a 40-μm strainer, suspended in 40% Percoll underlaid with 80% Percoll (GE Healthcare Life Sciences, UK) and centrifuged. The middle layer, an enriched population of leukocytes, was harvested. Cells were washed, resuspended in staining buffer consisting of 2% horse serum and 0.05% sodium azide, blocked with anti-mouse CD16/CD32 antibodies (clone 2.4G2) for 10 min or Fc receptor-binding inhibitor antibody for 20 min (Thermo Fisher Scientific, CA, USA), and then incubated with primary antibodies. Alternatively, following surface staining, cells were incubated with fixation-permeabilization buffer, washed with permeabilization buffer (Fixation/Permeabilization Solution Kit; BD Biosciences, CA, USA), and then incubated with antibodies against intracellular antigens. In cell population with low numbers (e.g., plasma cells), isotype controls were used for gating (see Supplementary Fig. S4). Samples were processed by a BD LSRFortessa (BD Biosciences) and analysed with FlowJo software (FlowJo, LLC, OR, USA). The antibodies used for flow cytometry are listed in Supplementary Table S3.

### Immunohistochemistry

Biopsied kidney tissues (4-μm thick sections) from healthy kidney donors and subjects without specific renal lesions were stained with anti-CD3, anti-CD68, and anti-CD14 antibodies, and counterstained with haematoxylin. Immunohistochemistry was conducted using a Ventana Benchmark XT automated staining system (Ventana Medical Systems, Tucson, AZ, USA). Positivity was quantified using pathology slide-viewing software (Aperio ImageScope; Leica Biosystems, Wetzlar, Germany). Twenty random fields were evaluated at 20 × magnification.

### Immunofluorescence staining

Mouse kidney tissues were cut into 300-μm sections using a needle blade, permeabilized and blocked in 0.1 M Tris containing 0.1% Triton X-100, 10% normal mouse serum, and 1% bovine serum albumin. Images were acquired using an LSM 710 microscope (Carl Zeiss, Cambridge, UK). The antibodies used for immunofluorescence are listed in Supplementary Table S1.

### Tissue clearing and immunofluorescence staining

Anesthetized mice were perfused with 50 ml of cold phosphate-buffered saline (PBS), followed by 50 ml of cold hydrogel monomer solution consisting of acrylamide (Sigma-Aldrich, MO, USA), VA-044 (Wako Pure Chemical Industries Ltd., Japan), and 4% paraformaldehyde Sigma-Aldrich) in PBS. Kidneys were removed and stored in fresh hydrogel monomer solution for 1–2 days at 4 °C, moved to 20 ml of fresh hydrogel monomer solution, and polymerized under de-gassed conditions at 37 °C for 2–3 h in an embedding device. The polymerized samples were placed in a chamber within the electrophoretic tissue clearing device (Crayon Technologies, South Korea). The reservoirs were filled with a clearing buffer consisting of 4% *w/v* sodium dodecyl sulfate (SDS), 50 mM lithium hydroxide (Junsei Chemical Co., Ltd., Japan), and 25 mM boric acid (Sigma-Aldrich) in dH_2_O. To preserve endogenous GFP proteins, samples were processed by a conducting device with 30 V at 30 °C for 16 h, and subsequent passive clearing with fresh 4% SDS buffer at 37 °C for 1–2 days with shaking. After clearing, tissues were washed in PBS for 12 h at 37 °C to remove residual SDS. The samples were incubated in PBS-Tween (0.5% Tween-20) solution with gentle shaking for 2 h at 37 °C, washed in PBS, and then incubated in blocking solution for 1 h at 37 °C. The staining of 3D images was performed by an electrotransport staining method using a C-stain device (Crayon Technologies). As a reflective index matching procedure, tissues were incubated in refractive index matching solution (88% [w/v] iohexol, 0.1% Tween-20 in PBS) for 0.5–2 h. Images were acquired using a Nikon C2si microscope (Nikon, Japan) with a Plan-Apochromat 10 × lens (numerical aperture = 0.5, working distance = 5.5 mm) with a 3 × zoom. Tissue was immersed in the refractive index matching solution during image acquisition. Three-dimensional rendering was performed using Imaris software (version 6.0; Bitplane AG, Switzerland).

### Statistical analysis

All analyses and calculations were performed using GraphPad Prism (version 7.0; GraphPad Software, Inc., CA, USA). The results are expressed as the mean ± standard deviation (or standard error of the mean in the Figures). Differences between groups were evaluated using the Student’s *t*-test. *P* < 0.05 was considered statistically significant.

## Supplementary information

Supplementary Information.

Supplementary Video 1.

Supplementary Video 2.

Supplementary Video 3.

Supplementary Video 4.

Supplementary Video 5.

## Data Availability

The datasets used or analysed during the current study can be obtained on reasonable request with the permission from the corresponding author.
