# Author Correction: Immune cell composition in normal human kidneys

**DOI:** 10.1038/s41598-021-83841-6

**Published:** 2021-02-16

**Authors:** Jun-Gyu Park, Myeongsu Na, Min-Gang Kim, Su Hwan Park, Hack June Lee, Dong Ki Kim, Cheol Kwak, Yon Su Kim, Sunghoe Chang, Kyung Chul Moon, Dong-Sup Lee, Seung Seok Han

**Affiliations:** 1 Department of Biomedical Sciences, Seoul National University College of Medicine, 103 Daehakro, Jongno-gu, Seoul, 03080 South Korea; 2 Department of Internal Medicine, Seoul National University College of Medicine, 103 Daehakro, Jongno-gu, Seoul, 03080 South Korea; 3 Department of Urology, Seoul National University College of Medicine, 103 Daehakro, Jongno-gu, Seoul, 03080 South Korea; 4 Department of Pathology, Seoul National University College of Medicine, 103 Daehakro, Jongno-gu, Seoul, 03080 South Korea

Correction to: *Scientific Reports* 10.1038/s41598-020-72821-x, published online 24 September 2020

This Article contains an error in Figure 6a, where the data shown does not correlate with the Article.

The correct Figure 6a appears below as Figure 1.

**Figure 1:**
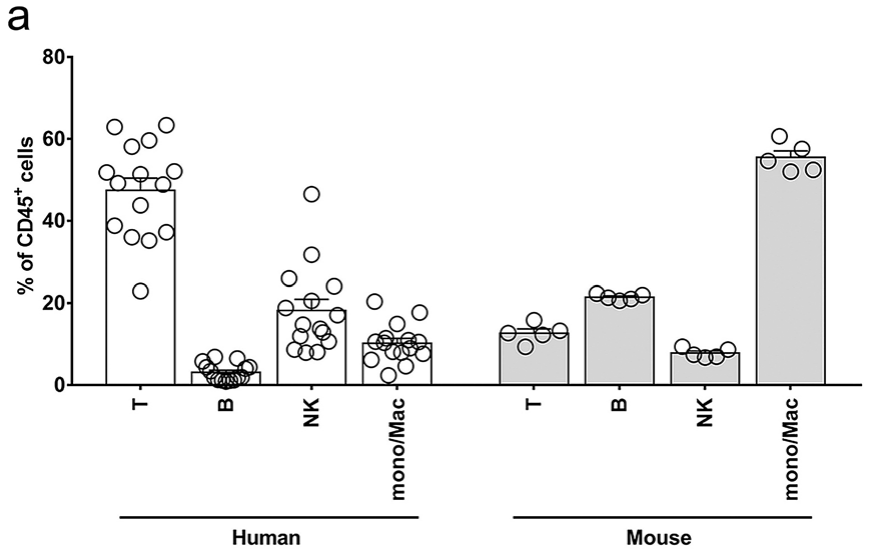
A correct version of Figure 6a.

