# Supplementary material for: Immune cell composition in normal human kidneys
Source: Sci Rep. 2020 Sep 24;10:15678. doi: 10.1038/s41598-020-72821-x (PMC7515917; doi:10.1038/s41598-020-72821-x)
Supplement: Supplementary file 1 — Supplementary Information. [file 41598_2020_72821_MOESM1_ESM.docx]

**Immune cell composition in normal human kidneys**

Jun-Gyu Park, Myeongsu Na, Min-Gang Kim, Su Hwan Park, Hack June Lee, Dong Ki Kim, Cheol Kwak, Yon Su Kim, Sunghoe Chang, Kyung Chul Moon, Dong-Sup Lee and Seung Seok Han

Tables S1-S3

Figures S1-S4

Legends of Videos S1-S2

Table S1. Baseline characteristics of study subjects for flow cytometry

| Variables | Total  (n = 15) |
| --- | --- |
| Age (years) | 69.1 ± 10.4 |
| Male sex (%) | 66.7 |
| Diabetes mellitus (%) | 13.3 |
| Hypertension (%) | 66.7 |
| Cancer (%) |  |
| Clear cell renal cell carcinoma | 63.6 |
| Papillary urothelial carcinoma | 36.4 |
| Blood urea nitrogen (mg/dL) | 16.6 ± 4.8 |
| Serum creatinine (mg/dL) | 1.0 ± 0.3 |
| Estimated glomerular filtration rate (ml/min/1.73 m^2^) | 73.6 ± 15.2 |
| Proteinuria (%) | 20.0 |
| Hematuria (%) | 20.0 |

Proteinuria and hematuria were defined as ≥ 1+ on a dipstick test.

Table S2. Histological findings of study subjects for flow cytometry

| Pathologic findings | Total  (n = 15) |
| --- | --- |
| Diabetic nephropathy (%) | 13.3 |
| Hypertensive nephropathy (%) | 6.7 |
| Tubular atrophy |  |
| < 25% | 93.3 |
| 25%–50% | 6.7 |
| > 50% | 0 |
| Interstitial fibrosis |  |
| < 25% | 6.7 |
| 25%–50% | 13.3 |
| > 50% | 0 |

Table S3. Antibodies used in this study

| Antibodies | Source | Clone |
| --- | --- | --- |
| Anti-human CD16-APC-Cy7 | BD Pharmingen | 3G8 |
| Anti-human CD11c-BV510 | BioLegend | S-HCL-3 |
| Anti-human CD11b-APC | BioLegend | ICRF44 |
| Anti-human CD64-PE | BioLegend | 10.1 |
| Anti-human CD33-BV711 | BD Horizon | WM53 |
| Anti-human CD14-PerCP-Cy5.5 | BioLegend | M5E2 |
| Anti-human CD14, unconjugated | Novus Biologicals | SC69-02 |
| Anti-human HLA-DR-PE-Cy7 | BD Biosciences | G46-6 |
| Anti-human CD45-BV786 | BD Horizon | HI30 |
| Anti-human CD45RA-PE-Cy7 | BioLegend | HI100 |
| Anti-human CD8-BV510 | BioLegend | Sk1 |
| Anti-human CD56-BV510 | BD OptiBuild | B159 |
| Anti-human CD69-FITC | BioLegend | FN50 |
| Anti-human CD25-APC | BioLegend | BC96 |
| Anti-human CD4-BV605 | BD Horizon | RPA-T4 |
| Anti-human CD19-PE-Cy7 | BD Biosciences | HIB19 |
| Anti-human CCR7-APC | BioLegend | G043H7 |
| Anti-human CD3-BV711 | BD Horizon | UCHT1 |
| Anti-human CD3, unconjugated | Abcam | PS1 |
| Anti-mouse/human CD3, unconjugated | Abcam | SP162 |
| Anti-human CD138-BV605 | BioLegend | MI15 |
| Anti-human TCRγ/δ-PerCP-Cy5.5 | BioLegend | B1 |
| Anti-human CD27-FITC | BD Pharmingen | M-T271 |
| Anti-human IgD-PE | BioLegend | IA6-2 |
| Anti-human Foxp3-PE | BioLegend | 206D |
| Anti-human CD38-APC | BD Pharmingen | HIT2 |
| Anti-human CD66b-FITC | BioLegend | G10F5 |
| Anti-human CD15-BV605 | BioLegend | W6D3 |
| Anti-human CD62L-PerCP-Cy5.5 | BioLegend | DREG-56 |
| Anti-human CD39-PerCP-Cy5.5 | BioLegend | A1 |
| Anti-human CD49a-PE | BioLegend | TS2/7 |
| Anti-human CD103-APC-Cy7 | BioLegend | BER-ACT9 |
| Anti-mouse CD69-BV785 | BioLegend | H1.2F3 |
| Anti-mouse CD3ε-BV711 | BioLegend | 145-2C11 |
| Anti-mouse CD62L-BV605 | BD Horizon | MEL-14 |
| Anti-mouse CD44-BV510 | BD Horizon | IM7 |
| Anti-mouse CD45.2-PerCP-Cy5.5 | BioLegend | 104 |
| Anti-mouse NK1.1-FITC | BD Pharmingen | PK136 |
| Anti-mouse CD19-PE-Cy7 | BioLegend | 6D5 |
| Anti-mouse TCRγ/δ-PE | BD Pharmingen | GL3 |
| Anti-mouse CD4-APC-Cy7 | BD Pharmingen | GK1.5 |
| Anti-mouse CD8-APC | BioLegend | 53-6.7 |
| Anti-mouse CD4-BV510 | BioLegend | RM4-5 |
| Anti-mouse CD25-FITC | BD Pharmingen | 7D4 |
| Anti-mouse FoxP3-PE | BD Pharmingen | MF23 |
| Anti-mouse/human CD11b-BV785 | BioLegend | M1/70 |
| Anti-mouse F4/80-BV711 | BD Horizon | T45-2342 |
| Anti-mouse I-A/I-E-BV605 | BD Horizon | M5/114.15.2 |
| Anti-mouse/rat XCR1-BV510 | BioLegend | ZET |
| Anti-mouse CD8-FITC | BD Pharmingen | 53-6.7 |
| Anti-mouse Ly6C-PE-Cy7 | BioLegend | HK1.4 |
| Anti-mouse CD45R/B220-PE | BD Pharmingen | RA3-6B2 |
| Anti-mouse CD11c-APC-Cy7 | BioLegend | N418 |
| Anti-mouse Ly6G-APC | eBioscience | 1A8-Ly6g |
| Anti-mouse CD16/CD32 | eBioscience | 93 |
| Anti-human Fc receptor-binding inhibitor | eBioscience | Polyclonal |
| Fixable Viability Stain 450 | BD Horizon |  |
| Fixable Viability Stain 780 | BD Horizon |  |
| Anti-rabbit IgG-HRP | Abcam | Polyclonal |
| Anti-mouse IgG-Alexa Fluor 546 | Thermo Fisher | Polyclonal |
| Anti-rabbit IgG-Alexa Fluor 647 | Thermo Fisher | Polyclonal |

Figure S1. Comparison of immune cell subsets in kidneys between male and female subjects (A) or subjects with and without kidney dysfunction (B).

(A)


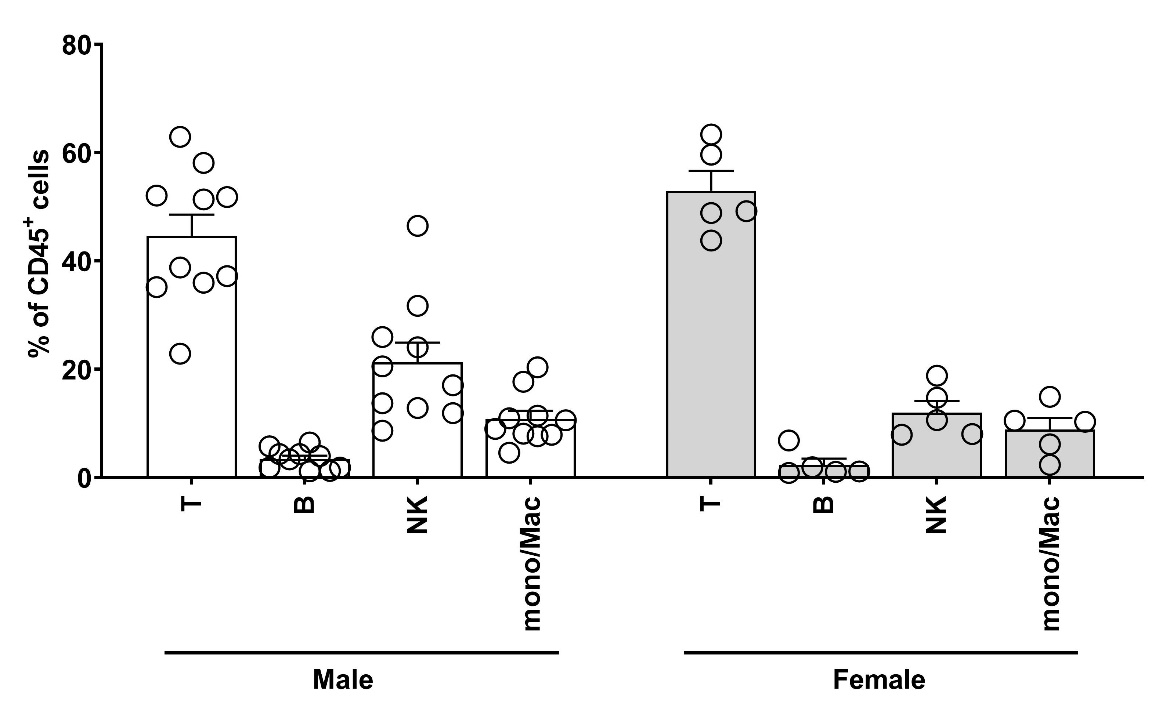


(B)


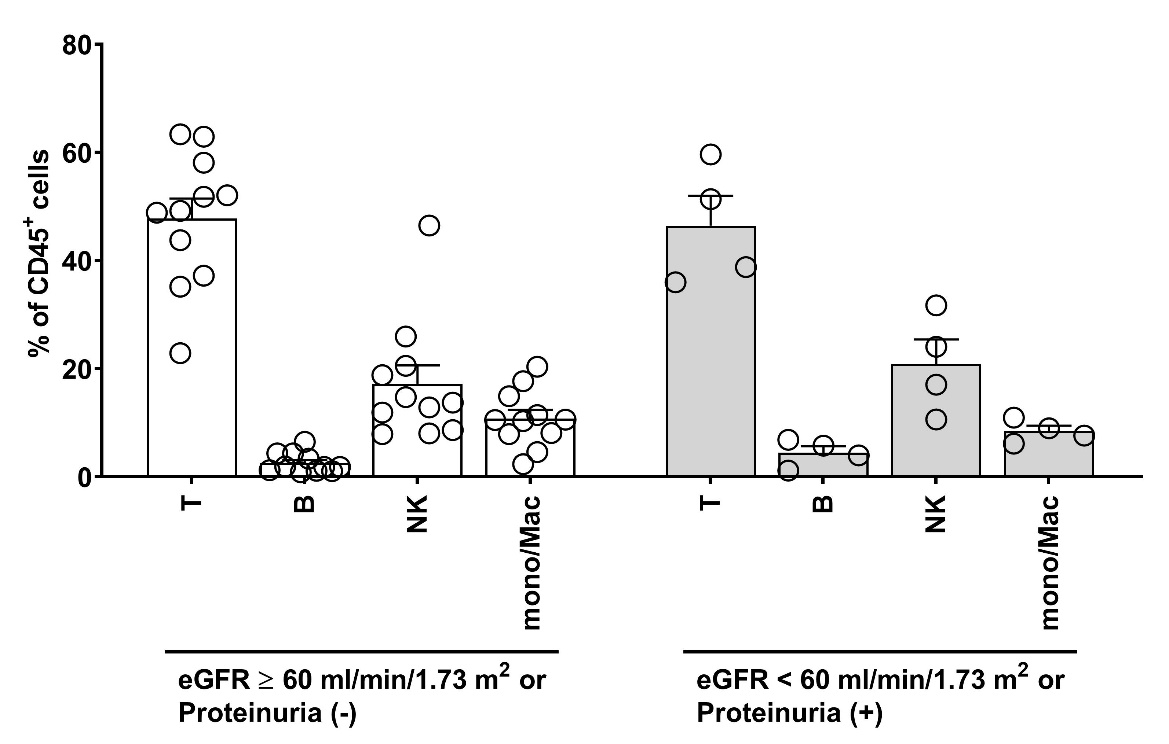


Figure S2. Representative images of staining for CD3, CD68, and CD14 in normal kidney sections from nephrectomised patients. Arrows indicate representative positive cells.


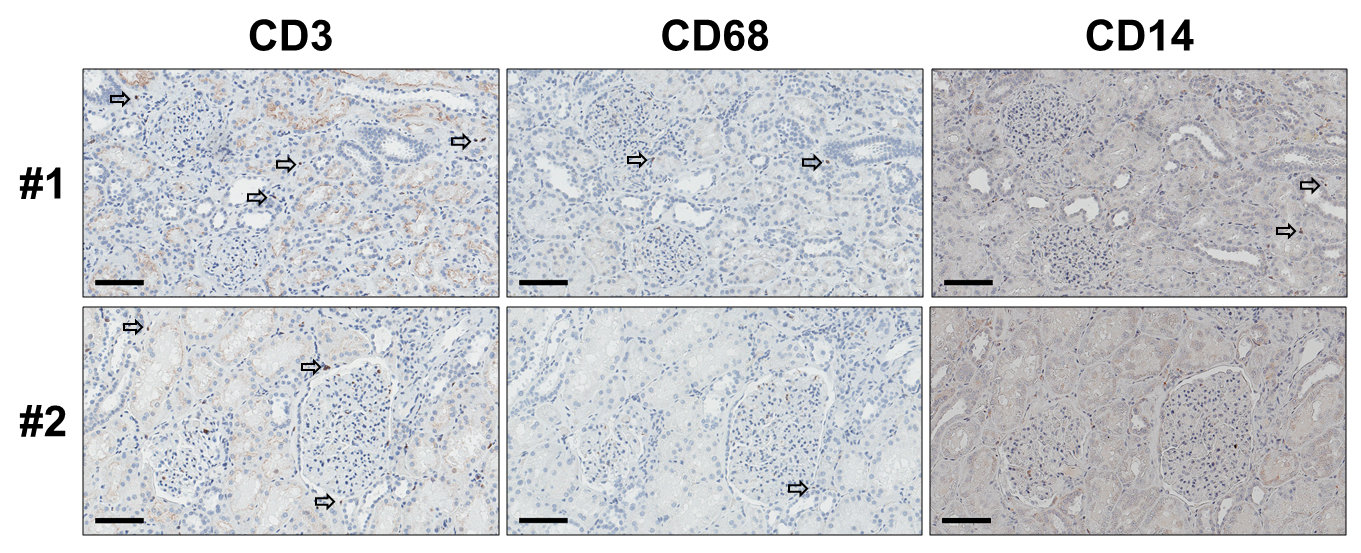


Figure S3. Proportions of T cells (A), myeloid cells (B), and other immune cell subsets (C) in 8-week-old BALB/c mice. TRM, resident memory T; rMac, kidney-resident macrophage; iMac, kidney-infiltrating macrophage; mono, monocyte; cDC, classical dendritic cell; pDC, plasmacytoid dendritic cell; Treg, regulatory T; gdT, gamma/delta T; NK, natural killer.

(A)


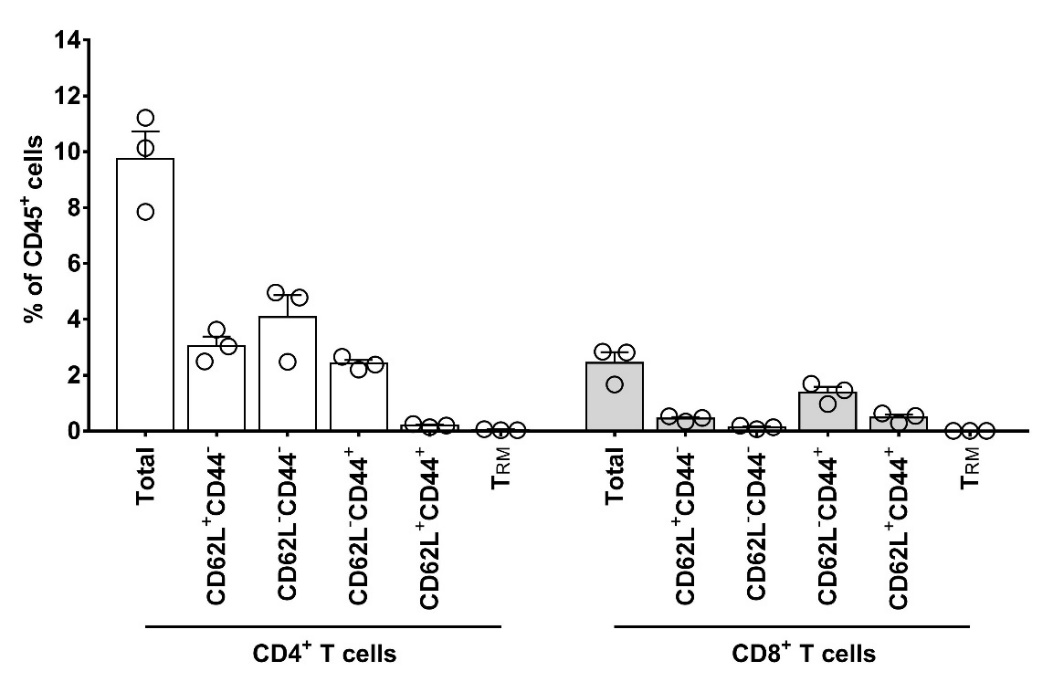


(B)


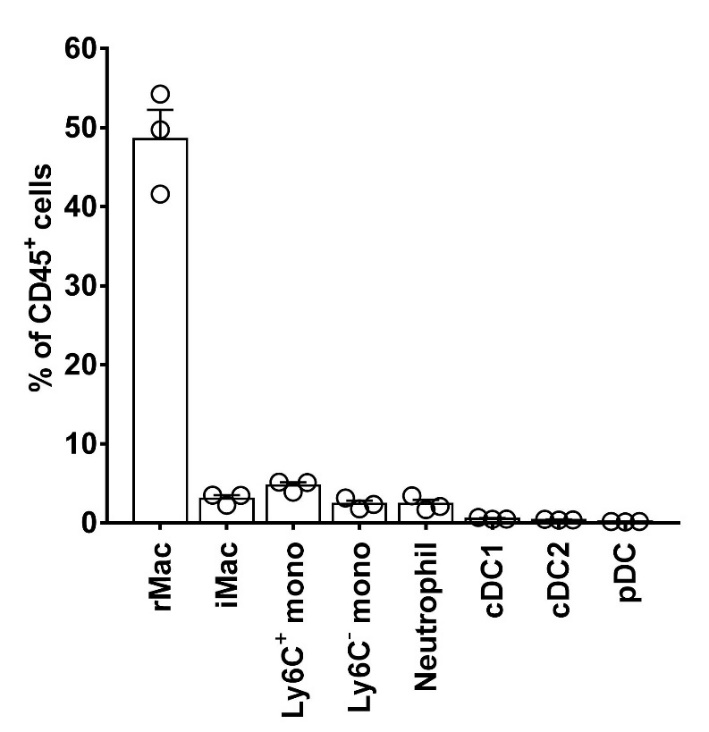


(C)


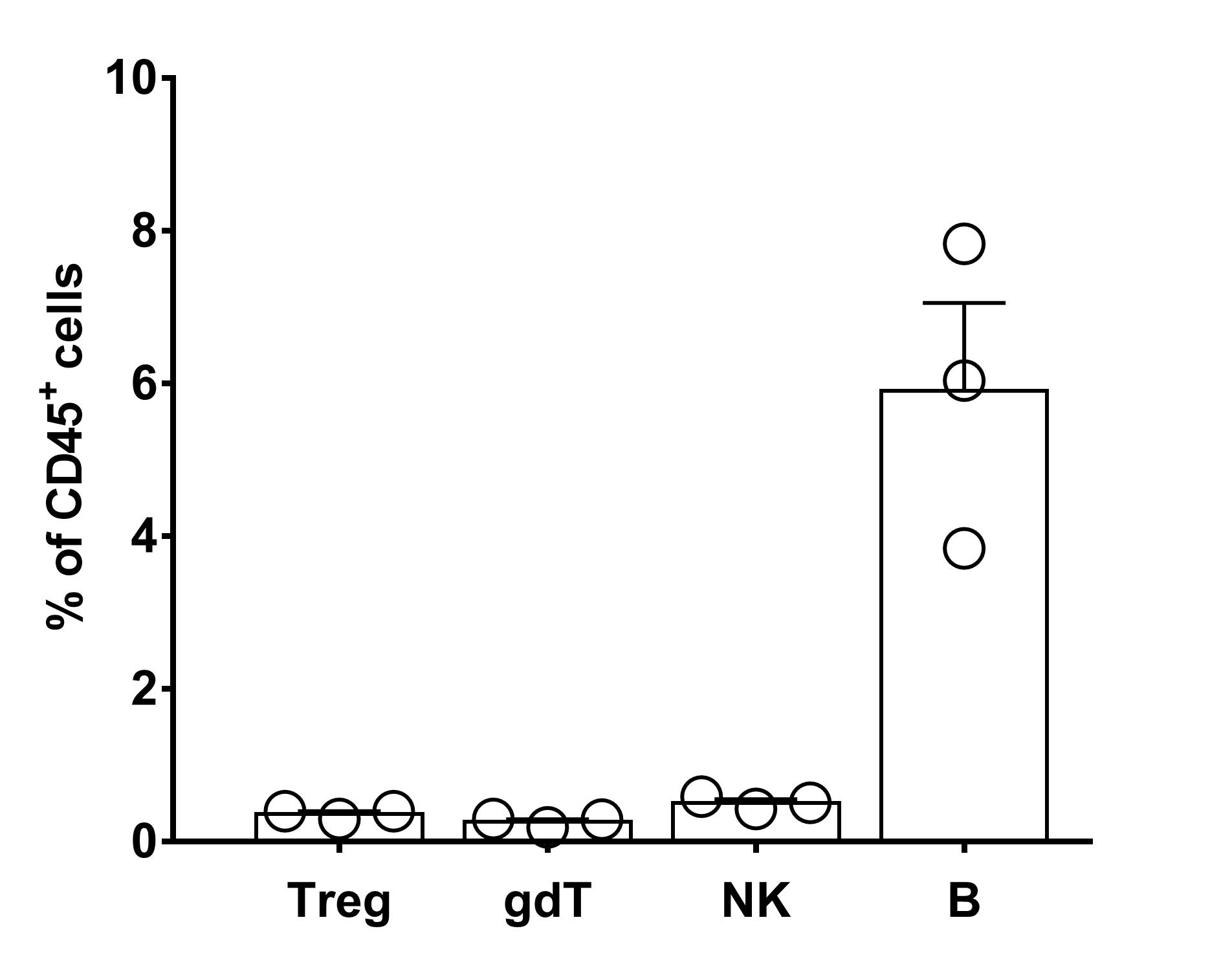


Figure S4. Flow cytometric staining of plasma cells.


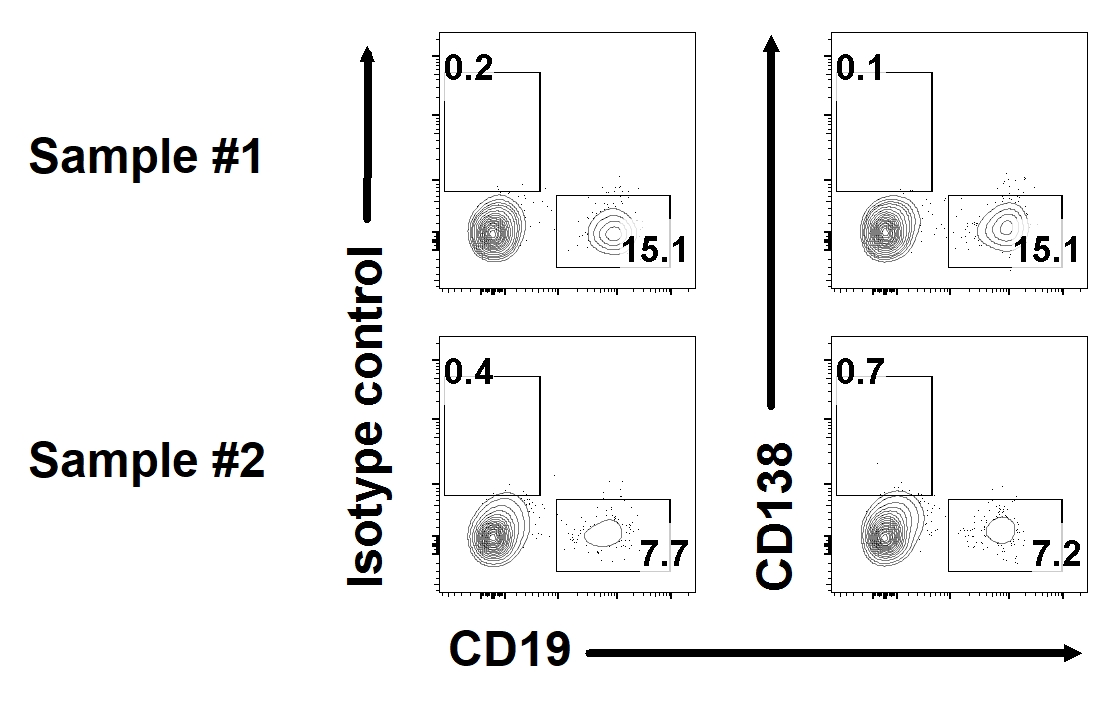


Video S1. 3D reconstruction of kidney tissue from healthy kidney donor, related to Figure 6B. (A) Full image. (B) Focused image, part 1. (C) Focused image, part 2. Magenta, CD3^+^ cells; green, CD14^+^ cells; blue, DAPI.

Video S2. 3D reconstruction of kidney cortex from *LysM*^Cre^–*ROSA*^mTmG^ mice, related to Figure 6C. (A) Cortex. (B) Medulla. GFP, lysozyme^+^ cells; red, CD3^+^ cells; white, other cells.
